# DEMENTIA CAREGIVER RE-ENTRY INITIATIVE: FINDING NEW DIRECTION AND PURPOSE AFTER CAREGIVING ENDS

**DOI:** 10.1093/geroni/igad104.2592

**Published:** 2023-12-21

**Authors:** Warren Wolfe, Sheryl Fairbanks

**Affiliations:** Freelance Journalist, Roseville, Minnesota, United States; University of Minnesota, Minneapolis, Minnesota, United States

## Abstract

Many former dementia caregivers struggle to find a “new normal” when the long and exhausting caregiving journey ends. Grieving eases, the estate is settled, and now what? Who am I after caregiving ends? Following 12 years of intense care for their parents, two with dementia, Sheryl Fairbanks and Warren Wolfe searched nationally for a program that might help them restart their lives. They found none. In 2016, they launched the Former Dementia Caregiver Re-Entry Initiative, which has served more than sixty people. At a kick-off meeting, former caregivers said their deepest needs were reestablishing relationships with family and friends and finding new life purpose. Typically, six to eight former caregivers meet twice a month – online since the COVID pandemic - and focus on moving forward. They share that these meetings give them a renewed purpose, deeper social connections, increased energy and a new sense of self-esteem. “My kids care. My friends care,” one member explained. “But nobody who hasn’t lived it really knows how hard that time was, and how hard it is to find a new path.” Four similar programs have started in Minnesota, The couple has written a book to encourage more. In 2022, Ms. Fairbanks and Mr. Wolfe became Community Leads in a University of Minnesota research project to see if Black former dementia caregivers have unmet needs and whether this model might help. Preliminary findings will be presented at the Addressing the Needs of African American Bereaved Dementia Caregivers symposium at this GSA annual meeting.

